# SmartPrompt Reminder Application Improves Everyday Task Completion and Reduces Inefficient Behaviors

**DOI:** 10.1093/geroni/igaa057.2908

**Published:** 2020-12-16

**Authors:** Hackett Katherine, Sarah Lehman, Ross Drivers, Matthew Ambrogi, Likhon Gomes, Chiu Tan, Tania Giovannetti

**Affiliations:** Temple University, Philadelphia, Pennsylvania, United States

## Abstract

The SmartPrompt phone-based reminder application was designed according to neuropsychological theory and pilot testing to facilitate everyday functioning. A laboratory-based pilot of ten participants with MCI and mild dementia showed significantly greater task completion with significantly fewer checking behaviors when using the SmartPrompt versus a control condition. Younger individuals and those who engaged in more checking behaviors completed more tasks in the control condition, but these relations were not significant when using the SmartPrompt. After 15 minutes of training, caregivers achieved near perfect scores on a SmartPrompt configuration quiz. Participant and caregiver usability ratings were strong, even though participants reported relatively low computer proficiency and neutral/unfavorable attitudes towards technology. Piloting informed modifications of the SmartPrompt to enhance personalization (e.g., customized alarms/rewards) and improved human-computer-interaction for in-home testing. Preliminary in-home test data on individually-owned smartphones and conclusions regarding barriers and facilitators to the effectiveness of the modified SmartPrompt will be discussed.

